# Activation of Innate Immunity by Bacterial Ligands of Toll-Like Receptors

**DOI:** 10.3389/fimmu.2014.00089

**Published:** 2014-03-05

**Authors:** Nelli K. Akhmatova, Nadezhda B. Egorova, Ekaterina A. Kurbatova, Elvin A. Akhmatov

**Affiliations:** ^1^Laboratory of Therapeutic Vaccines, I. I. Metchnikov Research Institute for Vaccines and Serum, Russian Academy of Medical Sciences, Moscow, Russia

**Keywords:** bacterial, poly-component p, vaccine, immunovac-VP-4, NALT, BALT, GALT, Toll-like receptors

## Abstract

Tγδ and B1 lymphocytes are essential components of the mucosal immune system, activated directly by different bacterial and viral ligands without additional costimulatory signals and preprocessing of other immune effectors. This ability enables the immune system to provide rapid protection against pathogens and contributes to the decoding mechanism of the sensitizing activity of mucosal antigens. The early interaction of these cells results in the production of antibodies of immunoglobulin M (IgM) and IgA isotypes, but not immunoglobulin E (IgE). We studied the subcutaneous, intranasal, and oral delivery as three major routes of potential entry for antigens of opportunistic microorganisms, using the immunomodulator Immunovac-VP-4, which is able to activate Tγδ and B1 lymphocytes. The subcutaneous and intranasal routes produced a significant increase of these cells in lymph nodes associated with the nasal cavity (NALT) and in those associated with bronchial tissue (BALT). The oral route significantly increased levels of these cells in the spleen, in NALT, BALT, and in nodes associated with the gut (GALT). We found that mucosal application of Immunovac-VP-4, which contains antigens of conditionally pathogenic microorganisms, in conjunction with the activation of Tγδ and B1, induces adaptive immune mechanisms not only in the lymphoid formations associated with the respiratory system and with GALT, but also in the spleen [increased expression of cluster of differentiation 3 (CD3), CD4, CD8, CD19, and CD25]. This indicates that there is migration of lymphoid cells from the regional lymph nodes and mucosal lymphoid tissues via the lymph and blood to distant organs, resulting in lymphoid development, and both local and systemic immunity. Mucosal application of Immunovac-VP-4 in mice potentiates the cytotoxic activity of NK cells in the NALT, BALT, and GALT. The highest cytotoxicity was observed in cells, derived from lymphoid tissue of the intestine after oral immunization. Although we found that cytokine production was increased by all three immunization routes, it was most intensive after subcutaneous injection. Our findings confirm that there is an intensive exchange of lymphocytes not only between lymphoid formations in the mucous membranes of the respiratory tract and of GALT, but also with the spleen, which means that if effective mucosal vaccines are developed, they can induce both local and systemic immunity.

## Introduction

Theoretical and experimental studies have shown that the main components of the mucosal immune system (MIS), determining the particular features of functional activity, are Tγδ lymphocytes and B1 cells in the epithelial mucosa, which have the ability to detect pathogen-associated molecular patterns (PAMPs) without costimulator signals and preliminary processing by other effectors cells (1–4). The MIS is also activated by other immunocompetent cells, which interact with Tγδ and B1 lymphocytes, resulting in stimulation of both local and systemic immunity in response to antigens (4, 5). Pattern-recognition receptors, Toll-like receptors (TLRs) recognize PAMPs of various pathogen classes, interact with cellular adaptive systems, and trigger the cascade of various signaling pathways to induce the complex of protective reactions (6–8).

The importance of such studies is obvious, yet the molecular and cellular mechanisms of innate and adaptive immunity activation in response to mucosal application of immunomodulators and vaccines have not been well investigated. Therefore, we conducted a study of mucosal immunity parameters, using the bacterial poly-component vaccine Immunovac-VP-4, which contains a wide panel of PAMPs. Various experimental models have shown the immunostimulatory effects of Immunovac-VP-4 (9, 10).

Bacterial poly-component vaccine Immunovac-VP-4 (Federal State Department, Science and Production Association, Microgene, Russia), created from the antigens of opportunistic microorganisms (*Klebsiella pneumoniae*, *Proteus vulgaris*, *Escherichia coli*, and *Staphylococcus aureus*), was designed for use in immunotherapy for chronic inflammatory and allergic diseases. The vaccine contains lipopolysaccharide associated with the outer-membrane proteins of the Gram-negative bacteria, peptidoglycan, teichoic acids, and lipoproteins of *S. aureus*, which are the ligands of TLRs 1/2, 4, 5/6, 9, revealed on cell line 293 [embryonic cells of human kidney, transformed by the E1 region of human adenovirus (11)].

## Materials and Methods

### Experimental animals

CBA mice, weighing 16–18 g, were obtained from the Scientific Center of Biomedical Technologies of the Russian Academy of Medical Science (Andreevka, Russia), and kept in the vivarium of the Metchnikov Research Institute for Vaccine and Sera at the Russian Academy of Medical Science. The housing, husbandry, and sacrificing conditions conformed to European Union guidelines for the care and use of laboratory animals.

### Mice immunization

For immunization, we used the bacterial poly-component vaccine Immunovac-VP-4, which contains antigens of conditionally pathogenic microorganisms (*E. coli, P. vulgaris, S. aureus, K. pneumoniae*).

Mucosal immunization was performed as follows: for intranasal immunization, the vaccine was applied in the nares in a single dose (an average of 500 μg in 30 μL). For oral immunization, the average single dose was 2000 μg/0.5 mL. Subcutaneously, the vaccine was administered twice within a 7-day interval at a dose of 200 μg (in 0.5 mL) for each injection. Twenty-four hours after the last vaccine administration, the mice were sacrificed using ether. The spleens, lymph nodes associated with the nasal cavity (NALT), nodes associated with bronchial tissue (BALT), and nodes associated with the gut (GALT) were analyzed and the number and phenotype of mononuclear cells expressing TLRs in the immunized mice were determined.

### Isolation of mononuclear leukocytes

Mononuclear leukocytes were obtained from lymphoid organs using Ficoll-Paque PLUS (GE Healthcare, USA) (12).

Quantitation of surface markers and TLRs was conducted using monoclonal antibodies (Caltag Laboratories, Carlsbad, CA, USA) against corresponding antigens, via flow cytometry (FACSCalibur). For the mononuclear lymphocytes from the spleens, lymph nodes, and intestinal nodes, we determined the level of expression for cluster of differentiation 3 (CD3), natural killer 1.1 (NK1.1), CD3/NK, CD4, CD25, CD4/CD25, CD8, CD19, major histocompatibility complex (MHC) class II, CD5.2, T-cell receptor Tγδ, TLR2, TLR4, and TLR9.

### Natural killer cell activity

Cytotoxic activity of mononuclear leukocytes from the lymphoid organs was determined using the NK-dependent tumor cell line K-562. Tumor cells (3 × 10^4^/mL) were incubated in culture medium (RPMI-1640, containing 2 mM glutamine, 5% v/v fetal calf serum, and 1% v/v benzylpenicillin sodium salt) with mononuclear leukocytes in a proportion of 1:5 in flat-bottomed 96-well plates for 18 h in a humidified incubator set at 37°C and 4% CO_2_. Next, vital dye MTT (Sigma) was added to the wells, the optical density at wavelength λ 540 nm was measured on a Multiscan MS (Labsystem, Helsinki, Finland), and the percentage of lysed tumor cells (cytotoxicity) was calculated.

### Subpopulations of lymphocytes

Subpopulations of lymphocytes were identified by flow cytometry using antibodies from Caltag Laboratories (Invitrogen, Carlsbad, CA, USA). The cells were washed in cold phosphate-buffered saline (PBS) and were stained with antibodies conjugated to fluorescein isothiocyanate and to phycoerythrin according to the manufacturer’s instructions. The suspension was then washed twice in cold PBS. The results were measured with a flow cytometer (FACSCalibur, Becton, Dickinson and Company, Franklin Lakes, NJ, USA). The cell population gate was determined by front and side light-scattering and cell size; there were 5000 cells per gate. Statistical analyses were performed with WinMDI (version 2.8; Microsoft, Redmond, WA, USA).

### Determination of cytokine levels

Cytokine levels in the blood sera of mice were determined by immunoassay (Bender MedSystems, San Diego, CA, USA) according to the manufacturer’s instructions.

### Statistical evaluation

All experiments were repeated at least three times. To study whether there is a general difference between three groups, first ANOVA test was performed, followed by Bonferroni *post hoc* test to compare specific groups or the Mann–Whitney *U t*-test was used to compare two individual groups. *P* values (*M* ± *m*) <0.05 were considered significant.

## Results

### Expression of TLRs

**Figure 1:**
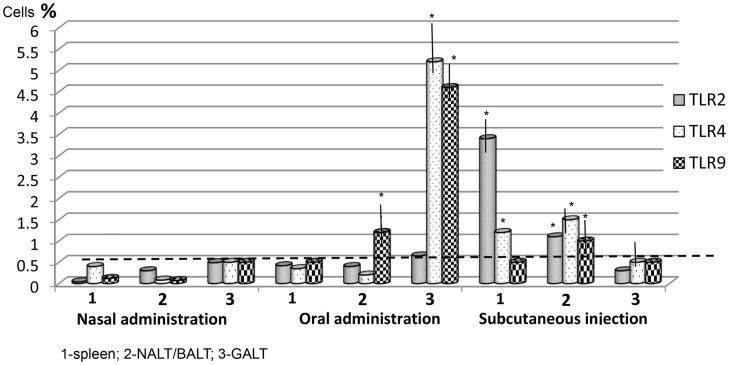
**The level of Toll-like receptors (TLRs) by route of vaccine administration, after one application**. The stippled line indicates the maximum levels of TLR expression in control (non-immunized) mice (*n* = 10 in each group). *Spleen*: nasal/oral admin. TLR2,3,9 *p* > 0.05; nasal/subcut. admin. TLR2 *p* < 0.01; TLR4 *p* < 0.05; TLR9 > 0.05, oral/subcut. admin. TLR2 *p* < 0.01; TLR4 *p* < 0.05. *NALT/BALT*: nasal/oral admin. TLR2,4 *p* > 0.05; TLR9 *p* < 0.05; nasal/subcut. admin. TLR2,3,4 *p* < 0.05; oral/subcut. admin. TLR2,4 *p* < 0.05, TLR9 *p* > 0.05. *GALT*: nasal/oral admin. TLR2 *p* > 0.05; TLR4,9 *p* < 0.001; nasal/subcut. admin. TLR2,3,4 *p* > 0.05; oral/subcut. admin. TLR2 *p* > 0.05, TLR4,9 *p* < 0.001. *p < 0.05 compared with control group (Mann–Whitney *U t*-test).

The level of TLR expression in the lymphoid organs of the control mice, based on data from three experiments, was low (0.01–0.5%) (Figure 1). It is important to note that in two trials, almost identical results were obtained, which may be considered normal values for non-immunized mice.

In the mice vaccinated once intranasally, the number of cells expressing TLRs did not increase significantly (Figure 1). After a single oral administration of the vaccine, there was a significant (*p* < 0.05) increase of TLR4 and TLR9 in the GALT (5.2 ± 0.5% and 4.6 ± 0.3%, respectively) and of TLR9 (1.2 ± 0.3%) in NALT–BALT. There were no significant changes in TLR levels in the spleen using this route of vaccine administration.

The distribution of TLRs for a single subcutaneous injection of 200 μg of the vaccine differed. The level of TLR2 (3.4 ± 0.3%) and TLR4 (1.2 ± 0.06%) in the spleen increased significantly (*p* < 0.05) to 1 ± 0.04% and 1.58 ± 0.06%, respectively. Also, the levels of TLR2, TLR4, and TLR9 in the NALT–BALT increased after subcutaneous vaccine administration. However, there were no changes in the level of TLRs in the GALT.

**Figure 2:**
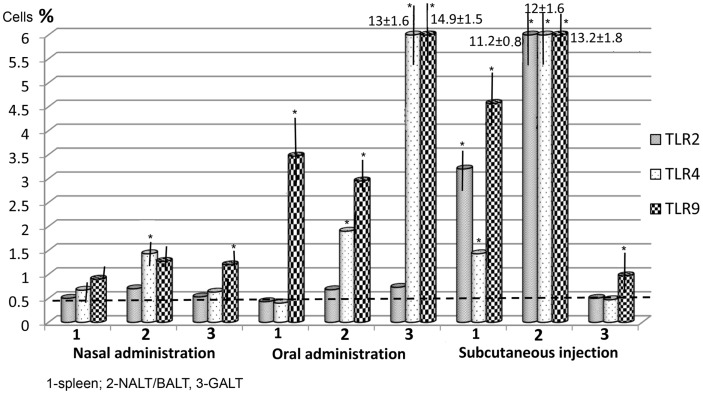
**The level of Toll-like receptors (TLRs) after three vaccine applications via the mucosa and after two subcutaneous injections**. The stippled line indicates the maximal levels of TLR expression in control (non-immunized) mice (*n* = 10 in each group). *Spleen*: nasal/oral admin. TLR2,3 *p* > 0.05, TLR9 *p* < 0.01; nasal/subcut. admin. TLR2 *p* < 0.01; TLR4 *p* < 0.05; TLR9 > 0.01, oral/subcut. admin. TLR2 *p* < 0.01; TLR4 *p* < 0.05, TLR9 *p* > 0.05. *NALT/BALT*: nasal/oral admin. TLR2 *p* > 0.05; TLR4 *p* < 0.05; TLR9 *p* < 0.01; nasal/subcut. admin. TLR2,3,4 *p* < 0.001; oral/subcut. admin. TLR2,4,9 *p* < 0.001. *GALT*: nasal/oral admin. TLR2 *p* > 0.05; TLR4,9 *p* < 0.001; nasal/subcut. admin. TLR2,3,4 *p* > 0.05; oral/subcut. admin. TLR2 *p* > 0.05, TLR4,9 *p* < 0.001. *p < 0.05 compared with control group (Mann–Whitney *U t*-test).

Toll-like receptors expression in tissue after three administrations was more significant. It was lowest in intranasal immunization, but TLR4 expression in the NALT–BALT increased to 1.43 ± 0.09%, while TLR9 expression in the NALT–BALT increased to 1.27 ± 0.17%, and TLR9 expression in the GALT increased to 1.2 ± 0.15% (Figure 2). After oral administration, the highest expression of TLR4 and TLR9 occurred in the GALT: 13 ± 1.62% and 14.9 ± 1.5%, respectively. In the NALT–BALT, the values were 1.9 ± 0.32% and 2.95 ± 0.65%, respectively. It is worth noting that the level of TLR9 also increased in the spleen to 3.47 ± 0.9%.

There were almost no changes in the level of TLR2 in the investigated organs following mucosal immunization. Subcutaneous immunization differed in several ways from mucosal immunization. First, the subcutaneous method increased the expression of TLR2, TLR4, and TLR9 not only in the spleen, but also in the NALT–BALT by 11.2–13.2%. Second, the level of expression of these three receptors was lowest in the intestinal mucosa, only slightly exceeding the values for non-immunized mice. Third, TLR2 was found in significant amounts only after subcutaneous administration.

### Immunophenotyping of the cells

The dynamics of lymphocyte immunophenotypes in mice spleens, NALT–BALT, and GALT was investigated. Table 1 presents data on the markers activated, by immunization route and tissue type, in control (non-immunized) mice, versus mice immunized with Immunovac-VP-4. Intranasal vaccination significantly increased the level of surface markers of effector cells in the lymph nodes of the NALT–BALT: Tγδ increased 32-fold (from 0.05 to 1.6%), B1 increased 8-fold (from 0.21 to 1.7%), and CD19 (from 0.63 to 25.2%) and CD4 (from 3.5 to 13.7%) increased 39-fold and 3.9-fold, respectively. In the spleen, the level of Tγδ increased 16-fold (from 0.17 ± 0.03% to 2.8 ± 0.02%) and the level of B1 (from 0.08 to 0.9%) increased 11-fold. In the GALT, all investigated values increased to lesser extent in comparison with those in the spleen and in the lymph nodes in the NALT–BALT. It is important to note that the mice received a relatively low dose of vaccine intranasally (total dose, 1500 μg), which was only 3.75 times higher than the subcutaneous dose (total dose, 400 μg).

**Table 1:** **Activation of leukocytes by immunization method and tissue type**.

Method of immunization	Level of leukocyte markers (*M* ± *m* %)^a^								
	Spleen			NALT–BALT			GALT		
	Marker	Intact	Immunized	Marker	Intact	Immunized	Marker	Intact	
Intranasally: three times	CD3	6.3 ± 0.61	47.4 ± 4.1 (7)	CD3	1.2 ± 0.6	7.6 ± 0.3 (6.4)	CD3	0.3 ± 0.16	2.7 ± 0.06 (9.0)
	CD3/NK	1.13 ± 0.65	3.63 ± 0.17 (3)	CD3/NK	0.4 ± 0.6	1.19 ± 0.16 (3)	CD4	0.34 ± 0.15	1.9 ± 0.02 (5.6)
	CD4	2.1 ± 1.2	58.6 ± 1.8 (28)	CD4	3.5 ± 0.8	13.7 ± 1.1 (3.9)	CD8	0.24 ± 0.09	3 ± 0.03 (12.5)
	CD4/CD25/Foxp3	0.8 ± 0.26	4.48 ± 1.1 (5.6)	MHCII	1.6 ± 0.4	72.0 ± 4.5 (45)	B1	0.15 ± 0.06	0.53 ± 0.03 (3.5)
	CD8	4.7 ± 0.74	34.2 ± 4.6 (7.3)	CD19	0.63 ± 0.2	25.2 ± 1.5 (39)			
	Tγδ	0.17 ± 0.02	2.8 ± 1.1 (16)	Tγδ	0.05 ± 0.02	1.6 ± 0.06 (32)			
	B1	0.08 ± 0.03	0.9 ± 0.02 (11.3)	B1	0.21 ± 0.15	1.7 ± 0.06 (8)			
Orally: three times	CD3/NK	1.13 ± 0.65	5.8 ± 0.6 (5.2)	CD3	1.2 ± 0.6	34 ± 5.5 (28)	CD3	0.3 ± 0.16	4.3 ± 0.9 (14)
	CD4	2.1 ± 0.2	13.3 ± 1.3 (6)	CD4	3.5 ± 0.8	40.1 ± 3.1 (11)	NK	0.7 ± 0.03	33.3 ± 1.2 (46)
	CD25	1.6 ± 0.65	12.6 ± 0.8 (7.8)	CD8	1.8 ± 0.08	31.9 ± 5.1 (17)	CD4	0.34 ± 0.15	7.3 ± 1.1 (21)
	CD4/CD25/Foxp3	0.8 ± 0.2	2.44 ± 0.04 (3)	CD19	0.6 ± 0.2	8.2 ± 0.6 (13)	CD25	0.7 ± 0.16	5.4 ± 0.5 (7.6)
	Tγδ	0.17 ± 0.02	1.43 ± 0.1 (3.7)	MHCII	1.6 ± 0.4	4.8 ± 0.8 (3)	CD8	0.24 ± 0.09	2.2 ± 0.15 (10)
	B1	0.08 ± 0.03	0.5 ± 0.06 (6.2)	Tγδ	0.05 ± 0.02	9.3 ± 1.8 (186)	CD19	0.4 ± 0.1	39.9 ± 0.8 (97)
				B1	0.21 ± 0.15	2 ± 0.3 (9.4)	CD3/NK	1.1 ± 0.16	13.7 ± 0.9 (12)
							MHCII	5.0 ± 0.9	51.7 ± 1.0 (10)
							Tγδ	0.2 ± 0.1	9.0 ± 0.8 (45)
							B1	0.15 ± 0.06	11.7 ± 0.9 (117)
Subcutaneously: two times				CD3	1.2 ± 0.6	10.4 ± 1.2 (8.8)	CD3	0.3 ± 0.16	2.4 ± 0.2 (8.8)
	CD4	2.1 ± 1.2	14.3 ± 2.26 (7)	CD4	3.5 ± 0.8	12.6 ± 1.0 (3.5)	CD4	0.34 ± 0.15	1.9 ± 0.16 (5.5)
	CD25	1.6 ± 0.15	5.6 ± 0.57 (3.5)	CD8	1.8 ± 0.	12.4 ± 1.9 (7)	CD8	0.24 ± 0.09	1.5 ± 0.2 (6)
	CD4/CD25/Foxp3	0.8 ± 0.26	2.8 ± 0.14 (3)	CD19	0.6 ± 0.02	2.3 ± 0.12 (3.5)	MHCII	5.16 ± 0.9	25.9 ± 0.7 (5)
	Tγδ	0.17 ± 0.02	5.3 ± 0.5 (31.2)	Tγδ	0.05 ± 0.02	12.0 ± 1.6 (240)	CD19	0.4 ± 0.1	1.75 ± 0.2 (4.3)
	B1	0.08 ± 0.03	4.9 ± 1.2 (62)	B1	0.2 ± 0.15	12.2 ± 1.6 (58)			

*^a^ Data are shown only for instances when marker levels increased by at least threefold*.

*BALT, broncho-associated lymphoid tissue; CD, cluster of differentiation; GALT, gut-associated lymphoid tissue; MHC, major histocompatibility complex; NALT, nasal-associated lymphoid tissue; NK, natural killer*.

After three oral applications of the vaccine, all investigated organs showed significant changes in cell numbers. In the GALT, the relative number of Tγδ lymphocytes increased 45-fold (from 0.2 to 9.0%) and the number of B1 lymphocytes increased 117-fold (from 0.15 to 11.7%). Only oral vaccination produced positive changes in the natural killer cell population, which increased 47.5-fold (from 0.7 to 33.3%). In addition, the number of cells with surface markers CD3, CD4, CD8, and CD19 increased significantly (11.5-, 17-, and 12.9-fold, respectively). In the NALT–BALT, the number of Tγδ lymphocytes increased significantly (from 0.05 to 9.3%), as did the number of B1 lymphocytes (9.5-fold), which is comparable with the results for intranasal vaccination. However, oral vaccination did not produce strong changes in the spleens of immunized mice, in contrast to intranasal vaccination: the percentage of CD4, CD25, Tγδ, and B1 increased only six- to eight-fold.

Subcutaneous vaccination led to a 240-fold increase in Tγδ (from 0.05 to 12.0%) lymphocytes in NALT–BALT and to a 31.2-fold increase of Tγδ lymphocytes (from 0.17 ± 0.03% to 5.3 ± 0.5%) in the spleen. Also, the high increase in the level of B1 lymphocytes in these organs compared with a much smaller increase in levels of other lymphocytes indicates that significant activation of the innate immune system cells in the spleen and lymph nodes in NALT–BALT was taking place. Intranasal vaccination produced less-dramatic changes in the intestine.

### Cytotoxic activity of NK cells

The cytotoxic effect of NK cells *in vitro* was assessed using the NK-dependent tumor cell line K-562 (Table 2). Cytotoxic activity significantly (*p* < 0.05) increased after only a single application of the vaccine. After subcutaneous injection, cytotoxicity increased, more in the spleen than in other organs, and was significantly (*p* < 0.05) higher compared with cytotoxicity in control mice and in the cells obtained from the lymphoid organs associated with the respiratory and gastrointestinal tracts. That trend was maintained after two subcutaneous injections of the antigens. Compared with mucosal immunization, three doses of oral immunization increased the cytotoxicity of spleen cells. In the NALT–BALT, there was a significant (*p* < 0.05) increase in cytotoxicity even after a single dose of the vaccine. Additional doses increased the cytotoxicity. In the GALT, there was a significant (*p* < 0.05) increase in cytotoxicity after both subcutaneous and oral vaccination. The highest degree of cytotoxicity occurred after oral immunization, in cells obtained from lymph formations: 32.6 ± 3.2% after a single dose and 75.4 ± 1.8% after three doses, which correlates with the data presented in Table 1 showing a significant (*p* < 0.05) increase in the percentage of NK cells after oral immunization alone.

**Table 2:** **Cytotoxic activity of mononuclear leukocytes against NK-dependent tumor cell line K562**.

Method of immunization with immunovac-VP-4	Cytotoxicity (*M* ± *m* %) by tissue type					
	Spleen		NALT and BALT		GALT	
	1^a^	2^b^	1^a^	2^b^	1^a^	2^b^
Intranasally	20.3 ± 1.3	12.0 ± 0.1	19.5 ± 2.5*	16.4 ± 0.72*	6.8 ± 0.4	8.4 ± 0.8*
Orally	19.9 ± 1.5	33.7 ± 1.0*	11.2 ± 0.7*	20.1 ± 0.8*	32.6 ± 3.2*	75.4 ± 1.8*
Subcutaneously	38.6 ± 3.1*	33.7 ± 0.9*	15.8 ± 1.5*	27.0 ± 1.0*	14.5 ± 0.7*	19.3 ± 2.2*
Control (non-immunized) mice	22.3 ± 2.8	18.3 ± 1.2	6.2 ± 0.8	7.7 ± 0.72	3.1 ± 0.4	2.0 ± 1.1

*^a^ Single dose of vaccine*.

*^b^ Three vaccine doses nasally or orally and two doses subcutaneously*.

*p < 0.05 compared with control (*t*-test).

*BALT, broncho-associated lymphoid tissue; GALT, gut-associated lymphoid tissue; NALT, nasal-associated lymphoid tissue; NK, natural killer*.

After repeated applications of the same antigens, the difference in results between immunization methods faded; however, the expression of NK and CD4–NK cells in intestinal lymph still persisted, but only after oral vaccination.

### Cytokine production

Cytokine levels were measured after mucosal immunization with a single dose of Immunovac-VP-4 versus levels after a single dose of subcutaneously administered vaccine for IL-1β, IL-6, IL-4, IL-10, IL-12, IL-5, tumor necrosis factor alpha (TNF-α), and IFN-γ in mice sera. Eight hours after injection, the level of the following cytokines significantly increased: IL-1β, IL-6, IL-12, and IL-5 (Table 3). However, their concentrations varied according to the route of vaccine administration. Cytokine production was most active after subcutaneous immunization, but mucosal immunization also presented significant results (*p* < 0.05). There was no significant difference in cytokine production between intranasal and oral immunization.

**Table 3:** **Level of cytokines in mice sera after single injection of immunovac-VP-4**.

Method of vaccine introduction	Level of cytokines, *M* ± *m* (pg/mL)							
	IL-1β	IL-6	IL-10	IL-12	IL-4	IL-5	IFN-γ	TNF
Oral	17.8 ± 0.6^a^	132 ± 16.3^a^	37.5 ± 2.8	12.5 ± 1.5^a^	5.8 ± 0.8	43.6 ± 1.5^a^ 1.7	7.5 ± 1.1	30.5 ± 3.1
Intranasal	18.2 ± 0.7^a^	115 ± 25.6^a^	39 ± 3.3	13.2 ± 1.2^a^	6.8 ± 0.7	55.5 ± 12.6^a^ 2.2	7.3 ± 0.5	33.7 ± 2.1
Subcutaneous	68.3 ± 3.2^a^	215 ± 15.8^a^	41.2 ± 4.5	38.6 ± 2.7^a^	5.5 ± 0.4	98.3 ± 9.8^a^ 3.8	36.6 ± 2.8^a^	36.6 ± 2.8
Control (non-immunized) mice	5.2 ± 0.5	46.8 ± 3.3	36.3 ± 3.8	5.2 ± 0.6	5.4 ± 0.6	25.7 ± 2.1	7.2 ± 0.5	28.3 ± 2.1

*^a^ The difference between the control and experimental groups; *p* < 0.05 (*t*-test)*.

*IL, interleukin; IFN, interferon*.

No difference in levels of IL-10, IL-4, or TNF-α was found among the various routes of vaccination. However, after subcutaneous injection of Immunovac-VP-4, the sera showed a fivefold increase in the level of IFN-γ compared with the control animals.

Multiple doses of Immunovac-VP-4 affected the expression of cytokines in mice sera by 24 h after the last application, significantly increasing the quantity of IL-1β, IL-5, IL-6, and IL-12 with all routes of vaccine administration. There was a significant increase in IFN-γ levels in that period even after a single subcutaneous injection of the vaccine.

## Discussion

After recognizing that pathogen-derived TLRs program the activation of antigen-presenting cells (APC) immediately after interaction with TLRs, Wolska et al. (13) showed that activation of TLR4 and TLR9 induces differentiation of Th1, while TLR2 activates Th2, which leads to the shift in the Th1–Th2 system to Th2.

The activation TLR4 and TLR9 in the absence of the expression of TLR2 is one of the stages of the mechanism of mucosal immunization that must be decoded, because at a sufficient dose, the technique provides better protection and significantly decreases the degree of immediate and delayed hypersensitivity in comparison with subcutaneous administration (14).

Subcutaneous administration produced a significant increase in TLR2, TLR4, and TLR9 levels in the spleen and in the NALT–BALT, but no significant change in the expression of TLRs within the intestinal mucosa. The level of receptors in the intestinal mucosa did not exceed 1% of the levels found with subcutaneous immunization, whereas oral immunization produced TLR4 and TLR9 increases of 14.9 ± 1.47% and 13 ± 1.62%, respectively. Intranasal vaccination also increased the level of TLR9.

It is noteworthy that oral immunization increased the expression of TLRs in all investigated organs, including the spleen. The participation of the spleen in this process indicates the development of not only local immunity, but also system immunity.

Our study produced the following findings:

- When mucosal vaccination is used, there is a prominent expression of TLR4 and TLR9 and an absence of the expression of TLR2.
- When oral vaccination is used, there is significant expression of TLRs in the GALT, NALT–BALT, and spleen.
- When intranasal vaccination was used in doses that induce a significant increase in the expression of TLRs, this occurred only in NALT–BALT, while subcutaneous administration resulted in increased levels of TLRs in the NALT/BALT and spleen. However, subcutaneous administration did not increase the level of TLRs in the GALT (an exception is TLR9). Similarly, subcutaneous injection does not induce the expression of TLR2 or TLR4 in the NALT–BALT, spleen, or in the lymphoid apparatus of the intestine; only TLR9 was expressed.

Hence, Immunovac-VP-4 evoked different pathways of cellular activation depending on the method of application: the subcutaneous route increased the expression of TLR4, TLR9, and TLR2, which led to further cellular differentiation in the direction of Th1 and to some degree in the direction of Th2. Expression of TLR4 and TLR9 after mucosal immunization predetermined the further development of the immune response toward Th1, inducing the production of interferon gamma (IFN-γ), thus suppressing the synthesis of IL-4 and the likelihood of further development of atopy.

An important feature of innate immunity activation is the increase of differential markers on lymphocytes, costimulatory molecules, and the APCs. This was revealed earlier in studies of subcutaneous use of Immunovac-VP-4. In our current investigation, we used different immunization methods to elucidate the features of MIS activation (15). Antigens of opportunistic bacteria, independent of the injection technique used, actively stimulate the innate immune system and result in the quick formation of non-specific resistance. Our investigation has shown that innate and adaptive immunity activation is triggered by recognition of pathogen-derived antigens by TLRs. Mucosal injection with Immunovac-VP-4 in the lymphoid organs associated with the mucosa and in the spleen engenders the expression of TLR4, and TLR9, with further possible differentiation of T-helper cells via the Th1 pathway. Activation of Th1 type cells accompanies IFN-γ production, inhibiting the synthesis of IL-4 and further development of atopy. It has been shown that subcutaneous injection of the same antigens also leads to a significant increase in levels of TLR2, leading to the further activation of Th1 and Th2. These data indicate that mucosal immunization may significantly decrease the degree of allergization by microorganisms (16).

The significance of TLR2 in sensitization that has recently been confirmed by several investigations (17–20), demonstrated that a polymorphism of the allele Ars7543708 TLR2 plays an important role in regulating the production of immunoglobulin E (IgE). The frequency of this mutant gene in the population, according to data from those studies, is not more than 5%, but the gene is present in 7.9% of patients with allergic diseases. The involvement of TLR2 in the development of an allergic reaction, and the associated production of IgE, suggests to investigate immune reactions resulting from the use of different bacterial and viral agonists of TLRs, and to determine the particular features of these administrations for immune activation for different routes of antigen application.

The decreased risk of allergic sensitization by different vaccines is the main advantage of mucosal vaccination over other methods, especially in children. The continued growth in the prevalence of allergic diseases and the lack of a safe, effective treatment for them poses a large problem worldwide. Thus, the development of mucosal vaccines and their widespread use may help decrease the burden of allergic pathology worldwide.

Apart from different levels of TLR expression, the immunophenotype of activated or recruited lymphocytes is also dependent on the method of vaccine application. Even a single dose of the vaccine Immunovac-VP-4 leads to increased levels of Tγδ and B1 lymphocytes in all mucosal methods of antigen introduction, and additional doses increase the levels even more. It is known that these cells are the major components of the MIS, characterized by the ability to recognize bacterial and viral ligands, without costimulatory signals and previous processing by other immune cells. This ability allows for rapid protection from different pathogens and makes it possible to decode how antigens introduced through mucosal immunization produce less sensitization than other methods, in such a way that interaction between these cells increases the production of antibodies IgM and IgA, but not of IgE.

Our findings confirm that mucosa-associated lymphoid tissue is the main systemic immunity organ (21–23). In view of the fact that the mucosa functions as the first line of protection against pathogens, Tγδ and B1 lymphocytes play the most prominent role in immunity, as they are capable to recognize the PAMPs of microorganisms without previous processing and presentation to MHC class II-restricted T lymphocytes.

All distinct vaccination routes showed some features in common. The increase of B1 and Tγδ lymphocytes was probably not only due to the vaccination route, but also to the character of the antigens used, which were processed in the cells without previous participation of MHC class II-restricted CD4 lymphocytes. It is noteworthy that the expression of NK cell markers involves the participation of MHC class II-restricted CD4 lymphocytes. We found that the expression of NK markers took place to a significant degree only when oral vaccination was used.

It is important to note that mucosal vaccination not only significantly increased lymphocyte activation in NALT–BALT and GALT, but also significantly increased the level of surface markers in the spleen, which is evidence of the development of not only mucosal immunity but also systemic immunity when this method was used. Only oral vaccination produced a significant increase in the number of NK cells, which are the main components of innate and adaptive immunity that correlate with maximum cytotoxicity in mononuclear leukocytes obtained from GALT. Although we found that cytokine production was increased by all three immunization routes, it was most intensive after subcutaneous injection.

In conclusion, our data demonstrate a high degree of activation of innate immunity effectors that is associated with the expression of TLRs, the emergence of a significant number of cells with differential surface marker molecules, the proliferation of the key effectors of mucosal immunity (Tγδ, B1, and NK cells), and an increase in cytotoxicity and cytokine production. In view of our findings, it is apparent that the development of a single-dose mucosal vaccine and associated vaccines should be a priority in contemporary vaccinology.

## Conflict of Interest Statement

The authors declare that the research was conducted in the absence of any commercial or financial relationships that could be construed as a potential conflict of interest.
